# 3D representation of Wnt and Frizzled gene expression patterns in the mouse embryo at embryonic day 11.5 (Ts19)

**DOI:** 10.1016/j.gep.2008.01.007

**Published:** 2008-05

**Authors:** Kristen Summerhurst, Margaret Stark, James Sharpe, Duncan Davidson, Paula Murphy

**Affiliations:** a Department of Zoology, School of Natural Sciences, Trinity College Dublin, College Green, Dublin, Ireland; b MRC Human Genetics Unit, Western General Hospital, Crewe Road, Edinburgh EH6 2XU, Scotland, United Kingdom; c ICREA, EMBL-CRG Systems Biology Unit, Centre for Genomic Regulation, UPF, Dr. Aiguader, 88, 08003 Barcelona, Spain

**Keywords:** Wnt, Fzd, OPT, Mouse embryo, 3D expression patterns, Comparative analysis

## Abstract

Wnt signalling is one of the fundamental cell communication systems operating in the embryo and the collection of 19 Wnt and 10 Frizzled (Fzd) receptor genes (in mouse and human) represent just part of a complex system to be unravelled. Here we present a spatially comprehensive set of data on the 3D distribution of Wnt and Fzd gene expression patterns at a carefully selected single stage of mouse development. Overviews and selected features of the patterns are presented and the full 3D data set, generated by fully described probes, is available to the research community through the Edinburgh Mouse Atlas of Gene Expression. In addition to being comprehensive, the data set has been generated and recorded in a consistent manner to facilitate comparisons between gene expression patterns with the capacity to generate matching virtual sections from the 3D representations for specific studies. Expression patterns in the left forelimb were selected for more detailed comparative description. In addition to confirming the previously published expression of these genes, our whole embryo and limb bud analyses significantly extend the data in terms of details of the patterns and the addition of previously undetected sites of expression. Our focussed analysis of expression domains in the limb, defined by just two gene families, reveals a surprisingly high degree of spatial complexity and underlines the enormous potential for local cellular interactions that exist within an emerging structure. This work also highlights the use of OPT to generate detailed high-quality, spatially complex expression data that is readily comparable between specimens and can be reviewed and reanalysed as required for specific studies. It represents a core set of data that will be extended with additional stages of development and through addition of potentially interacting genes and ultimately other cross-regulatory communication pathways operating in the embryo.

## 1 Results and discussion

### 1.1 The need for a comprehensive, integrated approach to gene expression analysis

Differentiation and morphogenesis are guided by a myriad of interactions of signalling molecules and signalling pathway components. One way in which signalling is controlled is through spatial and temporal restriction of the expression of genes encoding these molecules. To understand development we therefore need to follow the localisation of many gene products and this requires recording and retrieval of enormous amounts of data. A useful database to collect and display gene expression patterns has been compiled for the mouse by the Jackson Laboratories through Mouse Genome Informatics, where links to published and submitted data can be retrieved on a textual basis. However, published data from conventional gene expression analyses using whole-mount or section in situ hybridisation are limited and can often present only selected 2D images of the full 3D patterns that are not comparable across studies. For more complete knowledge it is necessary to record all sites of expression, as well as absence of expression, of developmentally important gene transcripts in time and 3D space.

Such a challenging task has become feasible with the development of the 3D imaging technique Optical Projection Tomography (OPT; Sharpe, 2003; Sharpe et al., 2002). OPT has been used in a variety of developing systems including human, mouse, chick, *Drosophila* and the plant *Arabdopsis thaliana* (Borello et al., 2006; DeLaurier et al., 2006; Kerwin et al., 2004; Lee et al., 2006; Lioubinski et al., 2006; McGurk et al., 2007; Miller et al., 2007; Sharpe et al., 2002). A particular advantage over other 3D imaging methods, such as confocal microscopy and Single Plane Illumination Microscopy (Huisken et al., 2004), is its ability to image the colourometric stains widely used for gene expression analyses. OPT is thus the most suitable 3D imaging method for simultaneously recording embryo morphology and gene expression patterns in mid-gestation vertebrate embryos.

To facilitate a comprehensive and integrative view of gene expression in the embryo, computing resources are required to store, retrieve and analyse large amounts of complex data. The Edinburgh Mouse Atlas of Gene Expression (EMAGE) (Baldock et al., 2003) pioneered the development of such tools and currently represents gene expression in time and space by mapping 2D expression data to reference 3D model embryos. Other initiatives generate and represent genome-wide 2D section expression data in query-able databases; EUREXPRESS (www.eurexpress.org), GENEPAINT (Visel et al., 2004) and the Allen Brain Atlas for adult mouse brain (Lein et al., 2007). By using OPT and advanced computing tools (Christiansen et al., 2006) 3D gene expression in the embryo can be represented in a database. Here we describe a focussed, comprehensive set of 3D data assembled in a consistent manner for inclusion in such a database. These data underline the enormous increase in information, of both expressing and non-expressing sites, represented when data are captured and analysed in 3D, the improved capacity for precise cross-gene comparison by being able to move through the 3D spatial representations and the added information in terms of aspects of the patterns (peaks and gradients in 3D) not readily appreciable from 2D sections.

### 1.2 Wnt and Frizzled gene expression patterns: generating and analysing the 3D data

Wnt signalling is one of the basic mechanisms of cell communication in all multicellular animals (Prud’homme et al., 2002; Schubert et al., 2000). It is important during development and homeostasis (reviewed in Cadigan and Nusse, 1997; Nusse, 2005), required for the availability of stem cells in adult tissues (Lowry et al., 2005) and implicated in the pathology of cancers (Major et al., 2007; Reya and Clevers, 2005). During development Wnt signalling is required for the establishment of most structures and systems, for example in the Central Nervous System (CNS) (e.g. Hall et al., 2000; Lee et al., 2000), somites (e.g. Galceran et al., 2001; Tajbakhsh et al., 1998), kidney (e.g. Kispert et al., 1998; Park et al., 2007) and many more. Elucidation of the diverse roles played by Wnt signalling during development is challenged by the complexity of the system with 19 known Wnt genes in the human and mouse, the products of which may interact with 10 Frizzled (Fzd) receptor gene products (reviewed in Huang and Klein, 2004), and/or an increasing number of additional/alternative receptors to stimulate at least three different types of pathway (reviewed in Gordon and Nusse, 2006). Furthermore the signal-receptor interaction can be modulated by a variety of extracellular proteins that can bind either to the ligands or receptors (reviewed in Kawano and Kypta, 2003). To reveal principles about Wnt pathway action in the developing embryo we need to consider groups of molecules working together in modules of activity (Hartwell et al., 1999) and here we begin to explore how data on distribution of a subset of the relevant molecules, Wnt and Frizzled gene transcripts, can be assembled in a manner that facilitates an integrated approach.

This study represents the starting point for a systematic collection of detailed 3D expression patterns across key stages of mouse embryonic development where data can be readily cross-compared and compiled in a searchable database. We present the first description of the data illustrating the quality and resolution achieved, the capacity for cross comparison and the limitations. To present a manageable amount of data we show only the expression of the genes encoding the Wnt signalling molecules (19 genes) and the Fzd receptors (10 genes) at a single stage of development; embryonic day (E) 11.5, Theiler stage (Ts) 19. For cross-comparison of the expression patterns we focus on the developing forelimb bud, an important site of Wnt regulated morphogenesis, to document and illustrate the potential of a comprehensive set of 3D data for these signalling molecule and receptor genes. The stage was chosen as a point just prior to overt cellular differentiation when the bud is composed of multiple territories that need to be defined in context of their gene expression patterns.

**Fig. 1:**
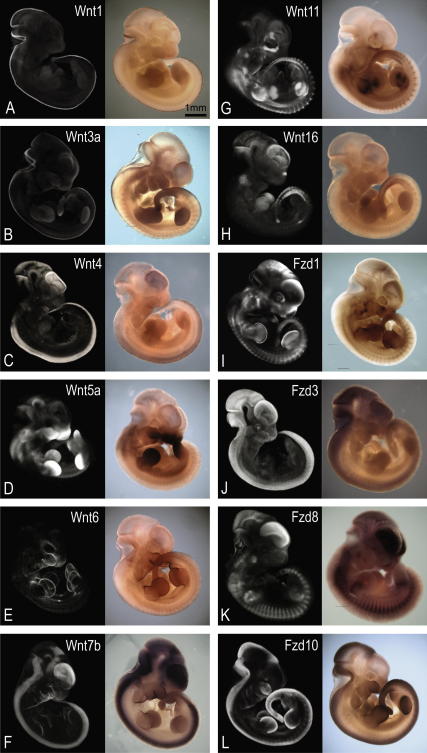
A selection of expression patterns of Wnt and Fzd genes viewed in whole embryos at Theiler stage (Ts)19. On the left are external views of volume representations of the 3D OPT scanned and reconstructed data. The right shows the corresponding whole-mount in situ hybridised embryo. The low level of staining has been optimised for maximum capture of the pattern by OPT. Areas of gene expression are seen as white/light grey in the volume representations. Note: some areas of apparent staining in raw specimens (e.g. limbs in H right) are due to viewing multiple layers of tissue and are not above background levels- this is evident in the 3D representation on the left. Scale bar as indicated.

Probes representing each of the 19 Wnt genes and 10 Fzd receptor genes were hybridised to a minimum of 10 Ts19 embryos in at least two independent experiments, however some more difficult patterns were generated in up to six experiments. A minimum of two specimens for each gene were scanned using OPT and the pattern reconstructed in 3D, and many were reconstructed up to 10 times to optimise parameters and check for variability. Movies showing 3D volume representations of the whole embryo pattern for each of the 29 genes can be viewed on a dedicated website; http://www.tcd.i/Zoology/research/WntPathway/. Fig. 1 shows still images of a selection of the patterns comparing an external view of the 3D OPT data (left) with the original photograph of the hybridised embryo (right). A range of staining intensities was generated (not shown) and tested. The staining intensities selected are deliberately low as staining which is too dark to transmit any light is not suitable for OPT imaging (acts as an optical barrier in multiple light paths and dampens autofluorescence, which is used to visualise the morphology of the specimen). However we have found that sites of low level expression, even if not clearly visible to the naked eye in the lightly stained original specimens are nevertheless picked-up by OPT and are clearly visible in the reconstructions; the sensitivity of the scanning technique on cleared tissue is greater than visual inspection of the original specimen and we have represented all of the expression sites visible in more intensely stained specimens.

We conclude that using carefully stage-matched specimens and selecting appropriate staining intensities, comparable 3D representations of expression patterns can be generated quickly. In light of our experience in this study, we recommend at least two independent hybridisations using 4–5 stage-matched embryos for each probe/stage, selecting a minimum of two structurally sound and appropriately stained embryos for scanning.

**Fig. 2:**
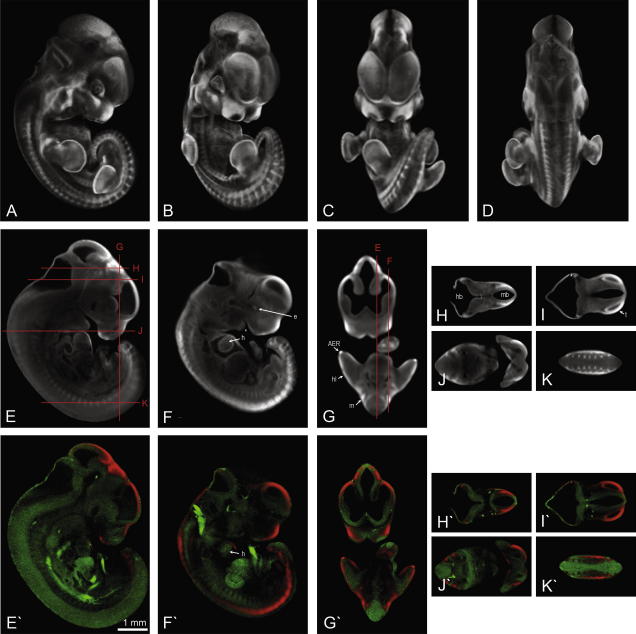
Fzd1 gene expression at Ts19 illustrating the various ways in which OPT generated 3D expression data can be viewed. (A–D) Still shots at various angles of a volume representation showing areas of expression in white/light grey. (E–K) Virtual sections taken through the 3D data in different planes indicated by red lines. (E′–K′) The same virtual sections following the merging of two OPT scans where the tissue and the gene expression were captured separately, the tissue pseudocoloured in green and the gene expression pseudocoloured in red. *Abbreviations:* AER, apical ectodermal ridge; e, eye; h, heart; hb, hindbrain; hl, hindlimb; m, myotome; mb, midbrain; t, telencephalon, ^∗^ indicates the 1st branchial arch, the 2nd arch is just posterior. Scale bar as indicated.

The images shown in Fig. 1 are external, projection views of the 3D data generated by OPT, mimicking the original whole-mount preparation but with the capacity to reveal deeper internal staining. The data can be viewed and analysed in any orientation or section plane as required. Fig. 2 illustrates multiple ways in which the Ts19 Fzd1 expression data can be viewed, showing external projection views of the 3D representations from different directions (Fig. 2A–D) and virtual sections in different planes. The tissue-level resolution (isotropic voxel dimension of ∼10 μm) allows expression to be assigned to a particular tissue or organ. Fig. 2E–K shows the raw data scanned using visible light representing the expression sites (high grey-level) against background tissue (low grey-level) and Fig. 2E′–K′ shows merged data from two-channel scans using visible light (pseudocoloured in red), showing the expression sites, and autofluorescence (pseudocoloured in green), showing tissue morphology. Merged data can also be visualised as external views of the 3D object (see Fig. 5, column A for examples). These methods emphasise different aspects of the expression pattern. Simple grey-level representation shows subtle details of the pattern such as differences in level of intensity and very fine spatial restriction that is sometimes obscured in images that use strong pseudocolouration. In contrast, pseudocoloured images often more clearly show the morphological context of expression.

Fig. 2 represents the data for a single gene (Fzd1) at a single stage of development (Ts19). Expression is evident in the dorsolateral mantle layer of the midbrain (Fig. 2H and H′); the lateral telencephalon (Fig. 2I and I′); a lateral stripe in the hindbrain just posterior to the midbrain hindbrain boundary (Fig. 2A, H and H′); the eye (Fig. 2A), localised in the anterior walls of the lens vesicle and ventral optic stalk (not shown); the frontonasal process around the nasal pits (Fig. 2B, F and F′), and complex patches in the anterior maxillary and posterior mandibular processes and 2nd branchial arch (Fig. 2A); the body wall around the heart (Fig. 2F′); the myotome and extending into the body wall (Fig. 2G′); a lateral body wall domain extending between the fore and hindlimbs (Fig. 2B); and in the limb, in the AER, patches in the limb mesenchyme, particularly anterior, and in patches in the dorsal aspect at the base of the limb (Fig. 5), particularly around the anterior forelimb (Fig. 2A). The selected sections show a subset of the full pattern visible in Fig. 2A–D; all possible sections and sites can be viewed in the 3D reconstruction available through EMAGE, for example using Edinburgh Mouse Atlas Project (EMAP) software (MA3DView and MAPaint http://genex.hgu.mrc.ac.uk/MouseAtlasCD/intro.html). This is the most comprehensive capture of the Fzd1 expression pattern. Previous expression data for Fzd1 showed expression in the thymus primordium at Ts20 (Bleul and Boehm, 2001), of which there was no indication in the present study yet at Ts19, and at earlier stages (Borello et al., 1999) in a number of sites corresponding to some of those reported here.

3D data at a similar level of detail and in database-ready form have been captured for the other 28 genes in the study (summarised in Table 1, available to view in 3D on http://www.tcd.i/Zoology/research/WntPathway/ and through EMAGE database entries). Tables 1 and 2 in Supplementary data list all of the noted sites of expression for each gene as well as previously published descriptions. The only gene for which no detectable expression above background levels was recorded was Fzd2. In all other cases our data extend previously published observations in terms of details of the pattern and newly recorded sites of expression.

### 1.3 Verification of the expression patterns

**Fig. 3:**
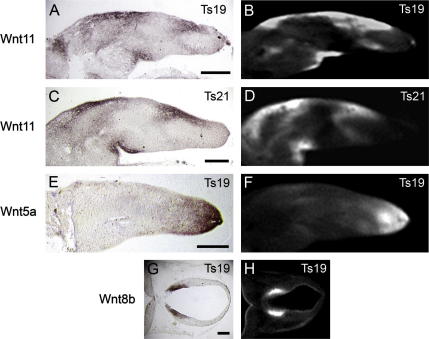
Comparison of in situ hybridisation to physical sections (A, C, E and G) with virtual sections of OPT scanned, whole-mount gene expression data (B, D, F and H). (A–D) Comparison of longitudinal sections of the limb bud showing Wnt11 expression at Ts19 (A and B) and Ts20 (C and D). (E and F) A comparison of Wnt5A expression in limb buds. (G and H) Compare the expression pattern of Wnt8B in sections of the brain (transverse section through the diencephalon). Scale bars = 250 μm.

Our ability to fully represent a gene expression pattern using OPT depends on our ability to fully capture that pattern using whole-mount in situ hybridisation. To ensure that we are seeing the full pattern we compared in situ hybridisation to embryo sections with virtual sections from 3D OPT generated data (Fig. 3). This was carried out for a variety of tissues and a number of stages. Fig. 3 shows limb expression of Wnt11, with a complex pattern within the mesenchyme and also in the AER at Ts19. At Ts20 there is a similar pattern in the mesenchyme but AER expression is no longer detected. Note that the sections are not identical across stages because of the difficulty of physically cutting identical planes; however sections are well matched between techniques (e.g. Fig. 3A with B) because of the flexibility to select any section of interest through the digital data. At both stages, the OPT generated data fully represents the data generated following hybridisation to sectioned embryos. Faithful OPT representation of the previously described graded pattern of Wnt5a in the limb (Gavin et al., 1990; Yamaguchi et al., 1999) is also shown (Fig. 3E and F). In the case of Wnt8b expression in the ventral diencephalon, the domain is two medial stripes either side of the floor plate (Fig. 3G and H). Here the OPT-generated image displays a clearer delineation of the expression domain than the cryo-sections, which can be distorted due to the physical cutting process, especially in early brain tissue with large vesicles. We have successfully used OPT to view expression in older specimens by dissecting part of the embryo, e.g. the limb or trunk region to view viscera up to Ts22 (not shown), similarly verifying penetration of probe and of the scanning method in each case. We have however had difficulty representing expression within very dense tissue such as the condensing skeletal elements by whole-mount procedures at later stages.

One limitation of the OPT data is that the resolution is not cellular so in some cases, particularly if the staining is strong, it is difficult to determine if expression in subectodermal mesenchyme extends into the ectoderm. In such cases, where the question is of importance to the system under study, it may be necessary to supplement OPT data with physical sections. For this reason we examined physical sections of whole-mount preparations to view expression of Wnt5a and Fzd10 in the distal limb. Physical sections showed that both genes are expressed in the AER at this stage (not shown); the literature reports a decline in the expression of Wnt5a in the AER from E11.5 (Gavin et al., 1990).

### 1.4 Overview of the patterns and selected observations

Table 1 summarises the data dividing the embryo into anatomical territories which are scored as expressing or not expressing (blank) each of the Wnt and Fzd genes. Despite the obvious limitation of such a textually based table to represent spatial patterns, it is clear that the majority of genes are expressed in multiple territories and each territory expresses a subset of the genes. The 3D data representations produced in this study and described here could be analysed to reveal and compare spatial distributions in each of these territories in detail. For example 21 of the 29 genes are expressed in the developing brain, 15 within the telencephalon. It is also interesting to note that a relatively large number of genes are expressed in the otic vesicle and the eye, each in specific and complementary territories within these developing sensory structures. One way to analyse the full complement of genes in a territory would be to select matching sections from the 3D representations for each expressed gene noted here (Miller et al., 2007). An example of such an analysis is performed below for the limb (Section 1.5). However in addition to simply comparing sections across specimens, in the longer-term the datasets could also be used for a full 3D comparison of gene expression patterns – a goal which relies on the spatially-complete datasets made feasible by OPT and presented here.

**Fig. 4:**
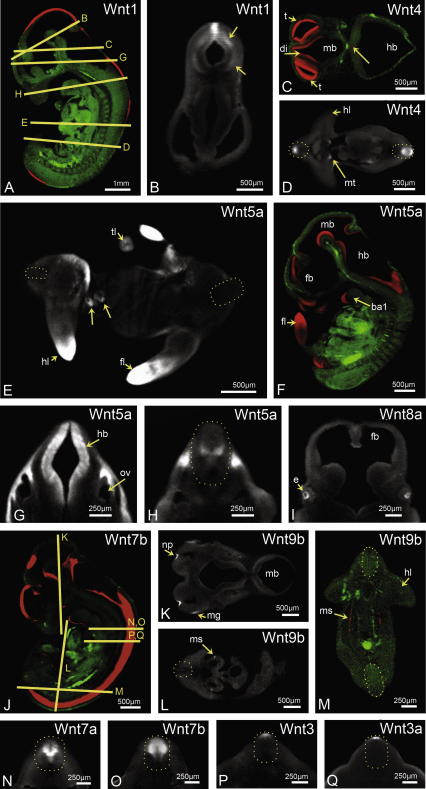
Selected Wnt expression sites in Ts19 embryos. The gene probe used is noted in each case. (A, F and J) Sagittal sections. (A and J) The orientation of section for each of the other images. (B) A transverse section through the midbrain/hindbrain boundary where Wnt 1 expression at this stage is high in the dorsal midline but absent from the ventral aspect and localised in marginal territories in basal and alar regions (arrows). The arrow in (C) indicates very localised expression of Wnt4 within the floor of the hindbrain. Arrows in (E) indicate asymmetric Wnt5a expression around the midgut in the umbilical hernia. (A, C, J, F and M) Views of merged (green = autofluorescence and red = brightfield showing expression) reconstructions. Neural tubes are outlined in (D, E, H and L–Q). *Abbreviations:* ba1, branchial arch 1; di, diencephalon; e, eye; fb, forebrain; fl, forelimb; hb, hindbrain; hl, hindlimb; mb, midbrain; mg, maxillonasal groove; ms, mesonephric duct; mt, metanephric mesenchyme; np, nasal pit; ov, otic vesicle; t, telencephalon; tl, tail. Scale bars as indicated.

Tables 1 and 2 in Supplementary data list sites of expression for each gene indicating newly described sites and added detail. The amount of pre-existing data varies but even for the most thoroughly described patterns additional aspects have emerged. For example the well documented domain of Wnt1 at the midbrain/hindbrain boundary (Bally-Cuif et al., 1992; Dymecki and Tomasiewicz, 1998; Wilkinson et al., 1987) is no longer throughout the dorsoventral extent of the neural tube at Ts19 but, while strong in the dorsal midline, is absent from the ventral floor and is restricted to the marginal zone in basal and alar territories (Fig. 4B). Wnt4 expression has been extensively described in the developing kidney (Stark et al., 1994) for example but much less well described in the CNS where we detected expression in different territories depending on the anterior–posterior level. It is throughout the ventricular zone of the forebrain and midbrain, strongest in the telencephalic vesicles and lowest in the midbrain (Figs. 4C and 1C). In the preotic hindbrain, expression is restricted to a very localised region of the ventral marginal floor plate (Fig. 4C, arrow). From just posterior to the otic vesicle expression was detected along the length of the neural tube, in different dorsoventral domains depending on the anterior–posterior level. From anterior to the base of the forelimb there are two peaks of expression in a broad ventricular territory, one in dorsal and one ventral to the midpoint. In more posterior positions a single dorsal domain is seen (Fig. 4D). Wnt5a expression has also been extensively described in the literature (Gavin et al., 1990; Grove et al., 1998; Yamaguchi et al., 1999) particularly in the limb, branchial arches and forebrain (Fig. 1D). In addition Fig. 4E shows asymmetric expression in the mesenchyme to one side of the midgut in the umbilical hernia. The CNS also showed additional spatial restriction of Wnt5a expression in a thick ventricular band in the ventral midbrain and a more restricted ventricular zone dorsally (Fig. 1D). Fine ventricular staining is seen throughout the midbrain/hindbrain boundary and expression becomes again more intense in a broad ventricular zone in the anterior hindbrain (Fig. 4F). At the level of the otic vesicle within the hindbrain there is a very broad ventricular domain, excluding the floor plate, with territories of different dorsoventral levels (Fig. 4G). In the most posterior part of the hindbrain Wnt5a is restricted to two patches just dorsal to the floor plate (Fig. 4H).

One generalisation that could be made from our survey is that Fzd genes tend to show more extensive and less tightly defined expression domains than Wnt genes (Fig. 1). Among Fzd receptor genes, Fzd8 and Fzd10 show particularly striking patterns; Fzd8 in the future sites of skeletal muscle and Fzd10 in the dorsal CNS and progress zones of the limb (Fig. 1).

Most of the Wnt and Fzd genes have multiple sites of expression in different systems, but there are notable exceptions. Wnt8b is specific to the forebrain where the pattern has been well described previously at Ts17 (Richardson et al., 1999; Theil et al., 2002). Here we show extra details of the pattern in 3D at Ts19 in the cortical hem and choroid invagination (http://www.tcd.i/Zoology/research/WntPathway/), and an additional domain in symmetrical stripes in the diencephalon (Fig. 3H), similar to expression of the human orthologue (Lako et al., 1998). The paralogous gene Wnt8a also shows very restricted expression, in this case in the lens epithelium of the developing eye (Fig. 4I) and otic vesicle. The Wnt9 paralogues also show restricted expression at this stage. Relatively little has been published on the expression of Wnt9 genes but Wnt9a has been reported in the limb mesenchyme in the position of perspective joints (Guo et al., 2004) and Wnt9b in the facial region (Lan et al., 2006). While we saw expression of Wnt9b in very localised points in the facial ectoderm, at the edges of the nasal pit and at the maxillonasal groove (Fig. 4K), no expression of Wnt9a was detected in the limb at this stage. Novel sites of expression however were detected for Wnt9a in the medial telencephalon, and the midbrain hindbrain boundary (not shown), and for Wnt9b along the entire length of the mesonephric duct (Fig. 4L and M).

**Fig. 5:**
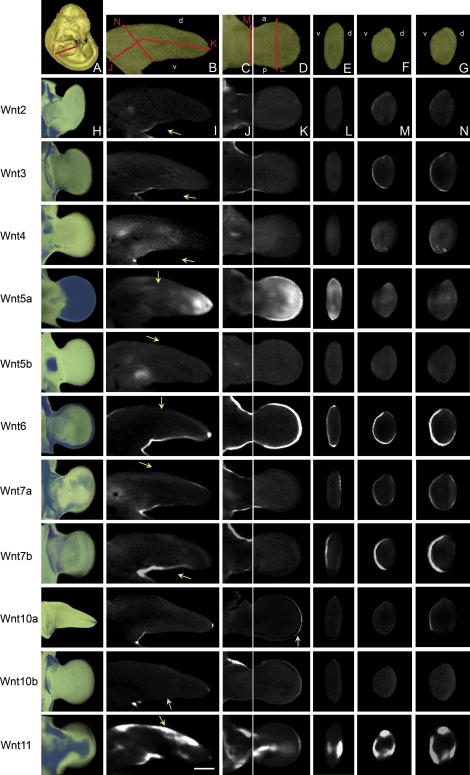

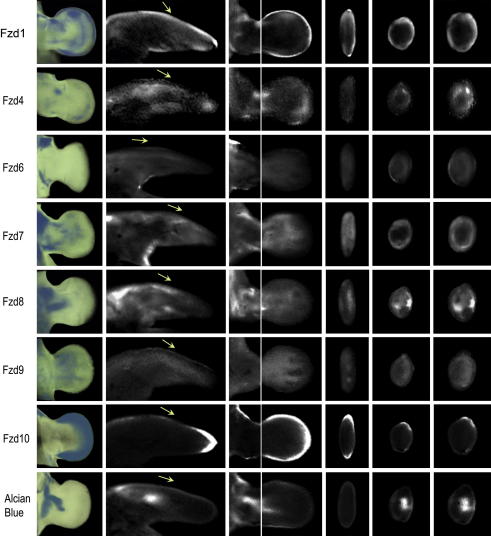
Comparison of Wnt and Fzd expression patterns and Alcian blue staining in the Ts19 mouse limb. The 11 Wnt and 7 Fzd genes that show localised expression within the limb at Ts19 are shown (Fzd3 shows general mesenchymal expression). The left column shows external views of volume representations of the limb bud where morphology (yellow) is merged with gene expression domains (blue). The arrows in the second panel of each row indicate the aspect viewed in the left column; note that these views were chosen to give the best overview of the pattern not for comparison. The top row shows a Ts19 model embryo for orientation. Columns under (B–G) show virtual sections of left forelimbs taken through comparable planes indicated by red lines: (B) Longitudinal through the dorsoventral axis; (C and D) Longitudinal through the aneterioposterior axis; (E–G) Transverse sections as indicated. Note that the longitudinal sections through the anterior–posterior axis were divided in two to accommodate the different angles of curvature of limb buds in different specimens. *Abbreviations:* d, dorsal; v, ventral; a, anterior; p, posterior. Scale bar = 250 μm.

There are notable similarities between other paralogous pairs of genes. Although distinctive, there are a number of similarities in the expression patterns of Wnt7a and Wnt7b in the CNS, limb ectoderm and otic vesicle. In cross section of the neural tube for example, Wnt7a is expressed in a broad domain at the midpoint tapering to ventricular toward the ventral (Fig. 4P) whereas Wnt7b is more dorsally expressed with elevated zones at the midpoint (Fig. 4Q). These genes are also expressed in different territories of the limb ectoderm (Fig. 5) and the telencephalon while in visceral organs, Wnt7a is expressed in the cystic primordium, whereas Wnt7b is in the epithelium of the lung bud. The Wnt3 genes are both expressed in the dorsal neural tube although Wnt3 in a more extensive domain than 3a in the dorsal midline (Fig. 4N and O). The Wnt5 genes are expressed in complementary patterns in the lingual swellings of the mandibular branchial arch, in the hindbrain, the limbs, around the foregut and stomach wall. The Wnt10 genes are both expressed in the AER of the limb with 10a staining less extensive along the A/P extent (Fig. 5). These similarities in the expression territories of paralogues may relate to their common origin from an ancestral gene and conservation of some aspects of the control elements. Further knowledge of the respective control regions will shed light on this interesting question.

### 1.5 Focus on the developing forelimb

The vertebrate limb is an excellent model of morphogenesis, beginning with the appearance of limb buds on the flank of the embryo from E9. Patterning is co-ordinated by the activities of at least three well described signalling centres, the Apical Ectodermal Ridge (AER), the polarising region and the dorsal ectoderm (reviewed in Johnson and Tabin, 1997). Such activity contributes to generating a cellular pattern for the later production of elements such as the digits, with species specific characteristic position, size and shape. Details of late morphogenesis, for example the positioning of joints in skeletal elements and the mechanisms that pattern the arrangement of muscles, tendons and ligaments remain largely unknown. Wnt signalling has been implicated in a number of aspects of limb development; in the initial positioning and outgrowth of the chick limb bud and later in patterning of the structure. In particular mouse mutations in two Wnt genes have shown their importance; Wnt5a in proximo-distal outgrowth (Yamaguchi et al., 1999) and Wnt7a in dorso/ventral patterning (Parr et al., 1998; Parr and McMahon, 1995; Yang and Niswander, 1995). However this is only part of the story: different Wnt gene transcripts and components of Wnt signalling pathways are dynamically localised within the developing limb, suggesting distinct roles for different Wnt genes or different regulatory scenarios for Wnt expression in different territories in the limb. Here we show how data on distribution of Wnt and Frizzled gene transcripts can be assembled in a manner that facilitates an integrated approach to considering groups of molecules working together to pattern territories in the developing limb at a time just prior to overt cellular differentiation.

Eighteen of the twenty-nine Wnt and Fzd genes are expressed in localised domains within the developing forelimb bud at Ts19; 11 Wnts and 7 Fzds (Tables 1 and 2). In addition Wnt16 is expressed in limb mesenchyme around the future skeletal elements in the positions of the future elbow and digit joints by Ts20 (not shown) and Fzd3 is expressed throughout the limb mesenchyme, slightly elevated in the proximal anterior region. 3D movies of the limb patterns are available at http://www.tcd.i/Zoology/research/WntPathway/, Table 2 summarises the expression in named limb subdomains and Fig. 5 shows comparisons of the raw expression data in the left forelimb. For comparison, Fig. 5 also shows the localisation of differentiating cartilage stained with Alcian blue. For an overview of the 3D patterns, the left column in Fig. 5 shows external views of volume representations of the 3D data with expression domains represented in blue. These are stills from the 3D movies (http://www.tcd.i/Zoology/research/WntPathway/). The angles viewed are different for each gene and were chosen to best show the pattern rather than for comparison across genes. The remaining columns show closely matched 2D virtual sections through the 3D data in two midpoint longitudinal planes (from dorsal to ventral and from anterior to posterior) and three transverse planes. While 2D views do not show all aspects of the 3D patterns, these show the possibility of comparing data across specimens generated in this way.

Aspects of the expression of several Wnt genes in the limb were previously undescribed. For example analysis of the complete set of serial virtual sections through the specimens revealed expression of Wnt2 and Wnt4 in localised patches of proximal limb mesenchyme (Fig. 5, column B) that were not visible from external views of whole-mount preparations. These domains appear to overlap each other, are adjacent to a patch of Wnt11 expression, do not coincide with Alcian blue stained tissue and are overlapping with expression of Fzd4, Fzd6, Fzd8, Fzd9 and Fzd3 and close to sites of Fzd1 and 7.

Wnt6 is clearly expressed in the AER (Fig. 5, columns B and C) in addition to surface ectoderm, where it is restricted to ventral ectoderm in the handplate (Fig. 5, column E) but extends more dorsally in the limb shaft (Fig. 5, columns F and G). Five Wnt genes are therefore expressed in the AER (5a, 6, 10a, 10b and 11) at this stage of development. Not all are expressed throughout the AER but have different boundaries of expression within the distal ectoderm with Wnt11 only detected in the most distal part of the AER, Wnt10a also more restricted than Wnt10b and skewed toward anterior. Three Fzd members show elevated expression in adjacent cells; Fzd1 and Fzd10 in the AER itself, Fzd1 also in adjacent surface ectoderm, Fzd4 and 10 in the progress zone with Fzd4 skewed toward the posterior, Fzd9 is at a low level in the mesenchyme of the future digit elements. Fzd3 is at a low level throughout the mesenchyme.

Most of the AER expressing Wnts are either expressed throughout the distal most limb (5a) or are ectoderm specific (10a, 10b and 6), except Wnt11 which is expressed in complex territories within the mesenchyme along its proximo-distal extent, not adjacent to the AER. From whole-mount views the expression appears to be at the “core” of the limb bud (Christiansen et al., 1995) but on section analysis it is clear that the expression is excluded from the very centre of the limb and is more subectodermal. A comparison with Alcian blue staining shows that the domains do not overlap with forming cartilage. Comparison with other Wnt and Fzd expression patterns shows that it overlaps partially with expression of Fzd1 and Fzd8.

In addition to the AER, Wnt10a and Wnt10b are also expressed in localised patches of ectoderm in the proximal limb. Both are expressed in ventral ectoderm at the base of the limb where Wnt10a extends more distally along the limb shaft. Wnt10b is most strongly expressed in an anterior patch on the ventral limb bud base, with a lower level patch just posterior to the midline (see 3D movie of the cropped limb http://www.tcd.i/Zoology/research/WntPathway/). The ectoderm at the base of the limb bud is in fact a site of expression of multiple Wnt and Fzd genes although the exact distribution and the foci of most intense patches of expression are different for each (Fig. 5, left column and 3D movies http://www.tcd.i/Zoology/research/WntPathway/). Wnts 2, 3, 4, 6, 7A, 7B, 10A and 10B, Fzd6 and Fzd7 are all expressed in the ventral ectoderm with Wnt7b and Wnt10b most intense toward the anterior aspect. Table 2 highlights also that multiple genes are expressed in a complex pattern within the mesenchyme that does not correlate to any know morphological territories. This is true for Wnt11, Wnt5a in the proximal bud, where it overlaps with Alcian blue staining, Fzd1, where it is most intense in patches in the anterior shaft, Fzd4, with three discrete patches in limb mesenchyme- in the posterior distal region of the progress zone and dorsal and ventral patches, anterior to the midline, surrounding the future skeletal elements (Alcian blue) in the limb shaft, Fzd8, with a pattern suggestive of the position of future muscle masses (a pattern somewhat complementary to Fzd4), Fzd6 and Fzd9, which show lower level but more extensive patterns, highest dorsally. Fzd7 shows particularly elevated expression in a ring in the proximal limb shaft, surrounding but significantly more peripheral than the Alcian blue stained cells.

One of the best-studied Wnt genes in the context of limb development is Wnt7a which has been shown to be important in signalling from the dorsal ectoderm and D/V patterning of the limb (Parr et al., 1998; Parr and McMahon, 1995; Yang and Niswander, 1995). Here we see that at Ts19 the dorsal ectoderm expression of Wnt7a is only found in the distal bud; in proximal regions expression is localised in ventral ectoderm. The paralogous gene Wnt7b is also expressed in the ventral ectoderm, extending more distally than 7A and is not expressed in the distal dorsal ectoderm so that transverse sections mid way along the handplate show Wnt7a in the dorsal and Wnt7b in the ventral ectoderm (Fig. 5, E column).

Wnt9a has been reported in the region of future skeletal joints in the mesenchyme of E11.5 mouse limbs (Guo et al., 2004) but we record no such expression at Ts19. This may be a staging issue since Guo et al. also report expression of Wnt16 in the territory of future joints at E11.5 which we record only very lightly stained in one specimen at Ts19 but clearly visible by Ts21. Nevertheless, we did not record any Wnt9a expression in the limbs of Ts20 embryos. It is important to note the dynamism of many of these patterns where we see differences in the expression of some genes among embryos that could be classified as Ts19. For example in very late Ts19 and Ts20 limbs Wnt10b and Wnt11 are no longer expressed in the AER. This underlines the importance of careful stage matching in comparative studies.

Two of the Fzd genes (2 and 5) did not show expression in the limb buds, while Fzd3 expression was not localised and Fzd7 and 9 showed widespread expression in the mesenchyme. Only Fzd9 and Fzd10 expression was previously reported at Ts19 in the mouse limb bud (Nunnally and Parr, 2004; Wang et al., 1999) while expression of Fzd1, Fzd3, Fzd6, Fzd7 and Fzd9 was briefly noted in the limb buds of earlier embryos (Borello et al., 1999). As reported by Wang et al. (1999), we observed Fzd9 expression in the mesenchyme of the future digits in the distal limb bud and additionally in dorsal mesenchyme of the limb shaft. We observed very similar expression of Fzd10 in the progress zone as was observed by Nunnally and Parr (2004), with expression distributed uniformly in a broad band of distal mesenchyme and ectoderm. In contrast expression analysis in the chick embryo has shown localised expression in the polarising region (Kawakami et al., 2000). It is possible that localised Wnt activity within the polarising region might be controlled at a level other than localisation of a specific receptor in the mouse. Alternatively, Wnt activity may be conveyed by a different Fzd. In this context we note elevated Fzd4 expression in the posterior region of distal limb mesenchyme. Fzd3 and Fzd7 transcripts are also present in this region although again not localised to the polarising region.

The selected descriptions compiled here reveal a number of new characteristics of Wnt and Fzd gene expression patterns. For example in the limb there are areas where multiple genes are expressed; hotspots of Wnt and Fzd gene transcription. These include very specific individual patterns within the surface ectoderm at the base of the limb, some highest in the anterior and complex patterns in the mesenchyme (Table 2). It is clear that the patterns do not relate simply to the known signalling centres in the limb supporting the concept of local interactions operating throughout the limb field. In some cases these interactions contribute to the demonstrated activities of signalling centres, for example Wnt7a in dorsal ectoderm (Parr and McMahon, 1995) and from the data here perhaps Fzd4 (or other more widely expressed Fzd genes) in the polarising region. Others may be involved in local interactions superimposed on or integrated with signals from previously defined signalling centres.

This study produced a volume of expression data that cannot be described entirely in a research paper. The full data set however can be viewed superficially at http://www.tcd.i/Zoology/research/WntPathway/ and is available through the EMAGE database.

### 1.6 Scope of the study

A number of large scale gene expression surveys have been undertaken to address our need to record the localisation of transcripts of developmental regulatory genes in the mouse, surveys of all transcription factor genes (Gray et al., 2004), genes expressed in the developing and mature retina (Blackshaw et al., 2004), the brain (Lein et al., 2007), the developing genitourinary tract (GUDMAP; Little et al., 2007), and data generated by the EUREXPRESS project (www.eurexpress.org). In contrast to such studies that examine a much larger set of genes in high-throughput screens, the focus of the effort here is to capture the data as completely as possible; complete in the sense of full spatial distribution and examination of all genes of a particular type. It is therefore possible to list from this work all sites of Wnt or Fzd gene expression in the Ts19 mouse embryo detectable by whole-mount in situ hybridisation and to identify any site where a gene or group of these genes is not expressed at a detectable level.

This represents a core set of data that can be built upon through the addition of other interacting gene transcripts such as those encoding other Wnt pathway components (currently under assembly), components of pathways that cross regulate Wnt signalling and ultimately linking into wider networks. Compiled with a much larger set of data in this way, this work will contribute to finding patterns that identify networks and modules of activity so that such modules can be examined functionally in the mouse and compared across species to gain an understanding of the evolution of molecular modules. The present study also allows cross gene comparison and has generated a resource which is suitable for a 3D data base that can be searched spatially. 3D recording allows aspects of the pattern, such as a very localised spot or the peak of a complex gradient, to be revealed. Such features cannot be fully represented in 2D, for example the concentration of Fzd1 transcripts in the anterior limb mesenchyme and the shape and distribution of patches of Wnt2, Wnt4 and Fzd4 in the limb (Fig. 5), because they cannot be appreciated from single 2D sections. Another example of such a feature is seen in the external view of Wnt7a limb expression (Fig. 5, Column A) where a slightly anterior skew within the dorsal ectoderm domain is discernable in the distal region, a feature not detectable in any of the sections.

Two limitations of in situ hybridisation however are important to note. Firstly the sensitivity of the technique may vary for different genes depending on the nature of the probes. The method cannot represent in absolute terms the respective levels of a particular transcript in different tissues or different transcripts in co-localised domains. Secondly, the dynamic range of the in situ visualisation methods currently used is such that it is not always possible to detect the lowest level of abundance of transcript without saturating detection at the highest level thus loosing, for example, an indication of expression gradients. The inevitable limits of sensitivity of the technique mean that the full domain of expression may actually extend beyond that recorded. However it should be noted that we do not know the functional relevance of a particular expression level so, while bearing this issue in mind the emphasis is on reliable recording (consistency across multiple specimens) of spatially-controlled patterns. In this study, we have made an effort to demonstrate the lowest levels of expression above background with selection of staining intensities that maximise capture of data across a range of levels. Certain features of the patterns, like the proximal limit of the Wnt5a graded expression in the limb, may vary slightly from specimen to specimen and it is therefore important to select the best specimens showing the clearest staining for 3D capture and entry into the database.

### 1.7 Mapping domains on to reference embryos for comparison and the potential for spatial searching of the data

As illustrated in Section 1.5, the 3D data can be compared across specimens by viewing stage and position matched virtual sections (Fig. 5). However computing tools can be used to map this data into reference embryos that provide a common spatial framework within which to compare patterns and make spatial searches of data from different experiments. In order to do this, it is necessary to distinguish signal from background and to capture a digital record of the former, for example by applying a threshold to the entire grey level image and selecting the domain that is above the threshold, as a signal domain. Using anatomical markers, the signal domain can be mapped to a standard embryo. Full details of this method have been published (Baldock et al., 2003) and are available at the EMAGE website www.genex.hgu.mrc.ac.uk, where mapping software is also available.

**Fig. 6:**
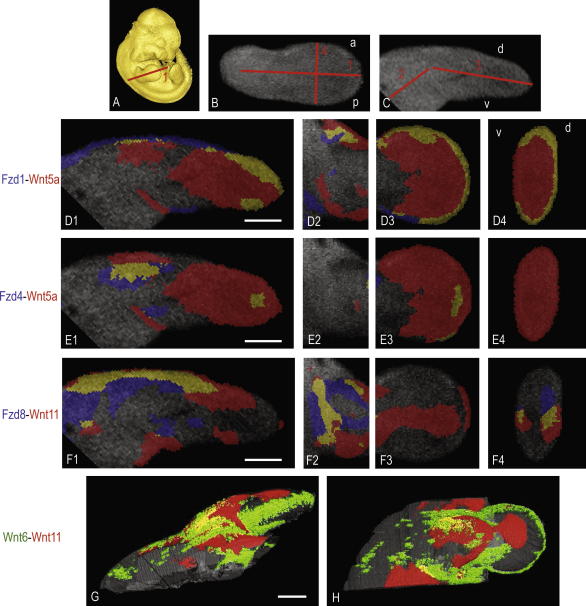
Examples of gene expression data mapped onto the left forelimb of reference embryos. (A) A surface representation of the reference Ts19 embryo with the orientation of sections D1, E1 and F1 shown. (B and C) Section planes 2, 3 and 4 on the cropped limb. (D–F) Each row shows the mapping of two genes noted on the left using the colour codes indicated. (G and H) 3D mapping of two genes indicated, this was achieved through serial mapping of matched 2D sections. Yellow indicates overlap.

Mapping signal domains in 3D is a complex problem not yet fully resolved. A simple alternative is to map signal from a section of the original 3D image to the corresponding section plane through the reference model. Examples of such ‘2D-mapped’ data are shown in Fig. 6 with three pairwise comparisons of genes selected as overlapping or complementary from analysis in Fig. 5. For example, Wnt5a in the distal mesenchyme and AER overlaps with Fzd1 through part of this domain, particularly in the periphery close to the ectoderm (Fig. 6D3 and D4). Note however that this is not uniform across the AP and DV axes with deeper overlap within the mesenchyme in patches in anterior handplate (Fig. 6D3) and shaft (Fig. 6D2) and absence of overlap in part of the posterior (Fig. 6D3) and ventral margins (Fig. 6D4). Wnt5a overlaps more extensively in the proximal limb with Fzd4 (Fig. 6E1) and in the distal limb overlap is restricted to a Fzd4 patch in the mesenchyme and a very localised subectodermal region at the posterior margin (Fig. 6E3). This posterior mesenchyme expression of Fzd4 is not reminiscent of the pattern of classic markers of the polarising region but may play a part in its activity. Fzd8 and Wnt11 patterns showed extensive overlap in the dorsal mesenchyme of the limb shaft (Fig. 6F1). Using existing EMAP computing tools it is relatively simple, though time consuming, to map data from one 3D representation to another via serial 2D virtual sections in this way (Fig. 6G and H).

The aim of producing a 3D dataset that can be spatially mapped and thus searched is important in order to maximise the benefits of capturing the full 3D data in a database. Only in this way can the spatial relationships within this complex data be explored. An impression of the limitation of a textual record is given in Table 1 which summarises the spatial distribution of the full gene set; this table can convey that a certain number of genes are expressed in a particular developing structure of the embryo but does not reveal their spatial relationship. In the presentation of the limb expression patterns here and the attempted descriptions, it is clear that many of the domains challenge textual representation. For example the expression of several genes in the limb mesenchyme, described as “complex mesenchyme” (Table 2), cannot be defined in terms of known anatomical domains. Therefore spatial recording of 3D domains within a common reference (through 3D mapping) is needed to permit sophisticated comparisons across potentially interacting genes. Here we present the original data for inclusion in such a 3D database which in addition to allowing direct analysis and detailed comparisons provides a frame for mapping, searching and retrieving the data based on spatial distribution.

## 2 Experimental procedures

### 2.1 Probes

Details of the probes used to generate the data presented for each of the genes is shown in Table 3 Supplementary data. Multiple probes were assayed for some of the genes showing variable sensitivity but no significant differences in the patterns.

### 2.2 Embryo collection

Embryos were collected from time-mated CD1 females on the morning of the 12th day following detection of a vaginal plug (E11.5). The embryos were precisely staged using Theiler criteria (Theiler, 1972), typically ranged between Ts18 and 20. Stage-matched embryos at mid-late Ts19 with a circular foot plate on the hindlimb and a characteristic oval shape in frontal view of the telencephalic vesicles were selected for the expression analysis presented here. The CD1 outbred strain was chosen for the establishment of a Wnt expression database in order to represent the normal expression pattern of these genes. No obvious variability in expression pattern was noted between specimens. It may be useful in the future to compare the expression of particular genes in inbred strains used in genetic manipulation studies to the CD1 pattern in the database, presently described.

### 2.3 In situ hybridisation (ISH)

The protocol used was largely as per Xu and Wilkinson (1998), optimised for OPT visualisation with the following changes: probe concentration of 1 μg/ml; post-hybridisation washes at 65 °C were 1× 5min in 50% formamide/5× SSC/0.5% CHAPS, then three serial 30 min washes in decreasing concentrations of formamide, SSC and CHAPS, culminating in 2× 30 min wash in 2× SSC/0.1% CHAPS and 2× 30 min in 0.2× SSC/0.1% CHAPS; preantibody blocking was in 3% blocking powder (Roche) in Maleic acid buffer; staining was carried out in the absence of Triton X-100. A minimum of two independent hybridisations with five embryos per probe were carried out for each gene where the expression patterns were very clear; for more difficult patterns up to six hybridisations were carried out often altering the probe being used. Each hybridisation included a sense control probe and Fgf8 (Crossley and Martin, 1995) as a standard by which to judge consistency across experiments. Since Fgf8 is expressed at different levels in different tissues, noting the time taken for staining to appear in the AER, the somites and the midbrian–hindbrain junction for example gave a good indication of the sensitivity of the experiment.

For the best OPT reconstruction data the intensity of colourometric stain should be moderate (as described in results). Also a low level of background staining of the tissue (so the tissue appears vaguely pink, Fig. 1) was found to be helpful in viewing OPT data captured in the visible channel alone as this allows the tissue context to be just visible when the full spectrum of grey level data is viewed (e.g. Fig. 2). The protocol was therefore optimised to produce such data. Staining components were diluted to 175 μg/ml 4-nitro blue tetrazolium chloride and 62.5 μg/ml 5-bromo-4-chloroindolyl-phosphate as a standard staining solution and staining was allowed to develop slowly with careful monitoring. For developing of very strong signals the above solution was diluted up to 1/10. Different intensities of staining were tested for each gene to ensure maximum capture of the data.

Embryos were physically sectioned (20–30 μm) using a Bright Model OTF Cryostat either prior to or following ISH. Embryos were fixed in 4% Paraformaldehyde overnight and embedded in 1.5% agarose, 5% sucrose. Trimmed blocks were equilibrated in 30% sucrose solution until they sank (usually overnight) and slowly frozen over a dry ice bath. About 20–30 μm sections were collected on BDH superfrost + slides and stored at −20 °C until hybridisation. In situ hybridisation to sections was carried out largely as described in Moorman et al. (2001) except proteinase K treatment was 10 μg/ml in 50 mM Tris, 5 mM EDTA for 5 min, the post-proteinase K fixation was in 4% paraformaldehyde, sections were dehydrated through an ethanol series prior to addition of probe, there was no pre-hybridisation and coverslips were used over the hybridisation solution, hybridisation was at 55 °C overnight, post-hybridisation washes were 2× 20–30 min in 50% formamide, 2× SSC, 65 °C; 3× 10 min in 2× SSC, 65 °C; 3× 10 min in 0.2× SSC, RT; 2× 10 min in TNT (100 mM Tris, pH 7.5, 150 mM NaCl, 1% Tween-20) at RT. The blocking solution for immunological detection of probe was Maleic acid buffer with 3% blocking powder (Roche).

Alcian blue staining was carried out as per Hogan et al. (1994).

### 2.4 OPT scanning and 3D reconstruction

After photographing the whole-mount data, at least two perfectly intact specimens from each hybridisation, representative of the externally visible pattern, were selected for OPT scanning. They were embedded in 1% low melting point agarose, dehydrated in MeOH overnight and cleared in benzyl benzoate/benzyl alcohol (1:2) (BABB) for at least 5 h (as previously described, Sharpe et al., 2002). Projection images of the specimens were captured in a prototype OPT scanner constructed at the MRC Human Genetics Unit, Edinburgh (Sharpe et al., 2002) and installed in the Zoology Department Trinity College Dublin. A Q imaging Retiga Exi camera was used to record images through a 360° rotation of the specimen viewed through a Leica MZ FLIII microscope with a plan 0.5× objective. Visible illumination was from a 20 W halogen lamp. At least two scans were performed for each specimen using visible light either with or without a 700 nm longpass filter, depending on staining intensity, to capture the expression pattern and under UV light using either a TXR filter (560/40 nm excitation, 610LP nm emission) or a GFP1 filter (425/60 nm excitation, 480 nm emission) to capture autofluorescence from the tissue to reconstruct embryo morphology. autofluorescence was found to be stronger using the GFP filter, however autofluorescence from blood was particularly strong under these conditions sometimes distorting the resulting 3D reconstruction. The raw data (400 projected images) from each of the scans were loaded onto a Linux workstation, reconstructed using a set of programmes provided by the Edinburgh Mouse Atlas Project (EMAP) and analysed using custom made software (MA3DView and MAPaint), again provided by EMAP. The isotropic voxel dimension of the objects is ∼10 μm.

To focus on expression in the limb, limbs were either dissected physically prior to scanning (not shown) or digitally cropped from full specimens for detailed comparison (Fig. 5). Expression patterns were compared crudely by viewing the volume rendered data externally or in detail by viewing matching section planes through stage-matched embryos. The section planes (described in Fig. 5) were carefully selected considering landmarks in all orientations within the 3D object. 2D mapping of data from different specimens was achieved using EMAP tools (MAPaint).

## Figures and Tables

**Table 1:** Summary of expression sites of all Wnt and Fzd genes in the whole embryo

*Abbreviations:* fn, frontonasal process; ba, branchial arch; nt, neural tube; drg, dorsal root ganglia.

**Table 2:** Summary of all Wnt and Fzd expression sites within the forelimb

Gene	Largely mesenchymal	Largely epithelial	Apical ectodermal ridge	Proximal epithelium	Prgress zone	Complex mesenchymal pattern	Handplate boundary
Wnt2	•			•			
Wnt3		•		•			•
Wnt4	•					•⁎⁎	
Wnt5a	•		•⁎		•		
Wnt5b	•			•			
Wnt6		•	•				
Wnt7a		•		•			
Wnt7b		•		•			
Wnt10a		•	•	•			
Wnt10b		•	•	•			
Wnt11	•		•			•	•
Fzd1	•	•	•			•	•
Fzd3	•						
Fzd4	•				•	•⁎⁎	•
Fzd6		•		•			
Fzd7	•					•	•
Fzd8	•					•⁎⁎	•
Fzd9	•					•	
Fzd10	•	•	•		•		

⁎ Gavin et al. (1990) report that AER expression of Wnt5a ends at E11.5. Our section in situ hybridisation results (Fig. 3) shows that AER expression persists until mid-Ts19.

⁎⁎ Associated with pre-muscle masses.

## Appendix A Supplementary data

Supplementary Table 1 Wnt gene expression sites at Theiler stage (Ts) 19. List of previously published sites and extended and novel descriptions in the present study. *n* = the number of specimens fully reconstructed in 3D (number of embryos hybridised from which the reconstructed specimens were selected as representative).

Supplementary Table 2 Fzd gene expression sites at Theiler stage (Ts) 19. List of previously published sites and extended and novel descriptions in the present study. *n* = the number of specimens fully reconstructed in 3D (number of embryos hybridised from which the reconstructed specimens were selected as representative).

Supplementary Table 3 Supplementary data: details of gene expression probes.
