# Supplementary material for: 3D representation of Wnt and Frizzled gene expression patterns in the mouse embryo at embryonic day 11.5 (Ts19)
Source: Gene Expr Patterns. 2008 May;8(5):331–48. doi: 10.1016/j.gep.2008.01.007 (PMC2452985; doi:10.1016/j.gep.2008.01.007)
Supplement: Supplementary Table 1 — Wnt gene expression sites at Theiler stage (Ts) 19. List of previously published sites and extended and novel descriptions in the present study. n = the number of specimens fully reconstructed in 3D (number of embryos hybridised from which the reconstructed specimens were selected as representative). [file mmc1.doc]

**Supplementary Data Table 1: Wnt gene expression sites at Theiler stage (Ts) 19**. List of previously published sites and extended and novel descriptions in the present study. n= the number of specimens fully reconstructed in 3D (number of embryos hybridised from which the reconstructed specimens were selected as representative).

|  | **Previously published sites**  **∅ Not confirmed here** | 🞲 Novel Sites◇ Extended details |
| --- | --- | --- |
| Wnt1  n=2 (6) | Dorsal midline throughout mesencephalon, diencephalon, neural tube, with gap in anterior hindbrain; midbrain-hindbrain boundary, ventral mesencephalon and diencephalon [1-3] | **◇**Not a full ring at the midbrain-hindbrain  boundary- strong in dorsal midline, absent in  ventral floor, localised to marginal zone in two  symmetrical sets of alar and basal territories. |
| Wnt2  n=8 (12) | Placenta at Ts17 [4], placenta at Ts19 [5], lung [6] | ×Branchial arches, distal patches on 1^st^ BA  and 2^nd^ BA, by Ts20 also patch on  pharangeal side of 2^nd^ BA. Also at Ts20,  three localised spots in maxillary process and  nasal septum  🞲Limb, mesenchymal patch, proximal core  🞲OV, most intense on medial and lateral sides  🞲Pericardium and body wall around heart,  complex pattern around atrium  **◇**Mesenchyme around lung buds  **◇**Placenta (precise extent in 3D)  ×Ventral body wall around viscera and  placenta  ×LPM bordering coelom from anterior to  forelimb to below forelimb, extends more  posteriorly in older specimens, to hindlimb  level by Ts20 |
| Wnt2b  n=8 (25) | Telencephalon, cortical hem, diencephalon, midbrain [7], optic cup (at Ts17, [7]),  **∅**lung, **∅** kidney, brain, endolymphatic duct, optic stalk and 1^st^ BA (at E12.5, [8]), gut (at Ts20, [9]), endolymphatic duct (OV) [10]) | ×Ventral part of neural tube, except very ventral  part of floor plate, from level of midway down  OV to base of forelimb, then reduced and  more ventrally restricted to level of somite 16.  **◇**Cortical hem and very fine dorsal midline  through diencephalon and mesencepahlon.  **◇**Lateral tips of optic cup and light in lens  vesicle.  ×Scleratome in upper trunk |
| Wnt3  n=4 (10) | Neural tube, brain, diencephalon, ectoderm, limb ectoderm, ([11], [12] midbrain-hindbrain, neuromere [13], rhombic lip- future cerebellum [14], oral epithelium [15] | **◇**Neural tube, dorsal domain, wider than  midline  **◇**Diencephalon, region with sharp anterior  boundary behind/underneath telencephalic  vesicles. Posterior boundary less sharp-  becomes lower level in posterior diencephalon  and more dorsally restricted in midbrain.  **◇**High in Rhombic lips of hindbrain  ×Ectoderm of Nasal process and distal  branchial arches  **◇** Limb ectoderm on proximal- ventral aspect  ×Otic vesicle, especially lateral side of the  endolymphatic diverticulum and cochlear duct  × Heart, wall of atrium |
| Wnt3a  n=3 (10) | Forebrain, telencephalon, cortical hem, neural tube ([7] [16] [11] [7] [17], tail [18] [19] | × Otic vesicle  ×Mesentery of midgut loop |
| Wnt4  n=4 (20) | Kidney, gonad, neural tube (not shown) [20], metanephric mesenchyme [21] limb mesenchyme [22], adrenal gland [23], oral epithelium, dental epithelium, head epithelium [15] gut (Ts20, [24]) | ×Telencephalon, diencephalon, cortical hem  and choroid plexus. Highest level and  throughout neuroepithelium in posterior  telencephalon, otherwise ventricular zone.  ×Ventricular zone of Mesencephalon, absent in  preotic hindbrain except for very localised  region of the ventral marginal floor plate.  **◇**Posterior to otic vesicle and throughout neural  tube; broad ventricular domain from dorsal to  ventral of midline, in most anterior part (until  just below level of forelimb) - two elevated  regions within domain, dorsal and ventral of  midline.  **◇**Branchial ectoderm particularly maxillary and  mandibular processes of 1^st^ BA and cleft  between 1^st^ and 2^nd^ BA. Ectoderm of  frontonasal process  **◇**Patches in forelimb mesenchyme and  proximoventral limb ectoderm  ×Localised in otic vesicle; highest in posterior  lateral wall (future semicircular canal)  **◇** Lining of midgut  **◇** Metanephric mesenchyme  **◇** Urogenital ridge |
| Wnt5a  n=6 (15) | Branchial arches, limbs, midbrain, frontal-nasal process, gut, genital tubercle ([25] [24] [26] [27] [28](not Ts19)), lung [29], mesenchyme under mammary region [30], cortical hem [7], pancreas ( at E11) [31], eye at Ts20 [32]  Ts21 cartilage condensation [33] | **◇**Broad ventricular in ventral half of the  mesencephalon. More restricted in ventricular  zone dorsally. Fine ventricular through  midbrain/hindbrain boundary  ×Broad ventricular in ventral hindbrain anterior  to the otic vesicle. In the hindbrain posterior to  the otic vesicle it is more extensively  expressed with territories of different levels  along the dorsoventral axis, floor plate  excluded. In the most posterior part of the  hindbrain it is restricted to two ventral patches  just dorsal to the floor plate  **◇**Localised in all branchial arch mesenchyme  ×Mesenchyme dorsolateral to OV  ×Optic cup  **◇**Mesenchyme around lung buds  ×Mesenchyme, polarised to one side of midgut  in umbilical hernia  **◇**Localised in mesenchyme of stomach wall-  anterior |
| Wnt5b  n=8 (15) | tail [34], eye at Ts20 [32], cartilage at Ts22 [33] | ×Floor of midbrain  ×Localised part of floor plate (ventral midline)  through neural tube from level of posterior otic  vesicle. Strongest in the posterior hindbrain  ×Lateral lingual swellings and posterior distal  aspect of mandibular part of 1^st^ BA  ×Medial margin of nasal pits  ×2 patches in ventral-proximal mesenchyme of  limb, with mild ectodermal staining on ventral  side of limb in area of posterior handplate  **◇**Optic cup  ×Mesenchyme surrounding lateral sides of  foregut in pharangeal region and extending  into entrance into 2^nd^ branchial pouch  ×Complex pattern in mesenchyme surrounding  foregut, level of 3^rd^ branchial arch  ×Intense spot on posterior aspect of aortic  pulmonary septum  ×Localised patches in mesenchyme of stomach  wall and around duodenum, complimentary to  Wnt5a  ×Cystic primordium  ×Distal tip of pancreatic lobe  ×Urogenital ridge  ×Scleratome / prevertebrae, particularly at and  anterior to forelimb bud |
| Wnt6  n=5 (15) | Genital ridge [35], oral epithelium [15], BA’s , ectoderm, mammary line [36], limb ectoderm and AER very visible, but not noted or described), ureter bud [37], dermomyotome at Ts15 [38] | **◇**Limb ectoderm including AER  ×Otic vesicle  ×Mesonephros  ×Mid-ventral point of urogenital sinus |
| Wnt7a  n=11 (35) | Eye at Ts17 and Ts20[32], limb surface ectoderm [26] [39] [55] (TS18), telencephalon [7] (TS20) | **◇**Complex pattern in ventral telencephalon,  diencephalon, mesencephalon,  metencephalon, rhomboncephalon, patches in  diencephalon.  ×Neural tube- transverse section shows broad  pattern at midpoint along dorsoventral axis, tapering toward ventral  ×Surface ectoderm of frontonasal region, BA’s,  limbs (see limb below), and posterior  embryo below forelimb level  **◇**Limb ectoderm, ventral in proximal limb and dorsal in distal limb, no AER  ×OV- ventricular surface- highest on medial  side.  ×Peritoneal-facing margin of midgut |
| Wnt7b  n=3 (8) | Eye (TS20, [32]), spinal cord (E10.5, [40]), telencephalon (Ts17, [41]). Knock in pattern[42], lung [29], pancreas [31] | **◇**Confirmation and extended detail in 3D of the  knock-in [42], showing expression in the eye,  spinal cord, BAs, and midbrain  ×Limb ectoderm on ventral side  ×Otic vesicle  ×To one side of the trachea  **◇**Epithelium of lung bud |
| Wnt8a  n=5 (12) | Hindbrain at Ts12 (rb4) [43], also [44], [32] mentions expression in eye lens but not shown | × OV  × Eye, throughout lens vesicle epithelium |
| Wnt8b  n=3 (20) | Ts17 forebrain [41, 45] | **◇**Only in forebrain, very specific and clear  pattern, cortical hem and separate patch in  choroid invagination- not continuous, medial  anterior part of telencephalic vesicles  ×Two stripes on either side of the floor of the  diencephalon- located medially, away from  ventricular zone and marginal zone (similar  pattern reported for human at Carnegie stage  15-21[46]) |
| Wnt9a  n=6 (25) | **∅** Limb mesenchyme [22] | ×Medial part of telencephalic vesicles  ×Dorsal midline stripe in midbrain/hindbrain  boundary- more extensive on midbrain side  ×OV, extensive but symmetric pattern of  elevation |
| Wnt9b  n=6 (20) | Face [47] | **◇**Facial ectoderm: very localised points on the  ectoderm-edges of nasal pit and at maxillo-  nasal groove, and 1^st^ BA  ×Mesonephric duct along entire length |
| Wnt10a  n=13 (35) | **∅** Eye (Ts20, [48], mandible primordia, tongue [49], AER seems down regulated at E11.5 compared to 10.5, [50] | **◇**Patch on mandibular component of 1^st^ BA  ×Ectoderm at posterior aspect of ventral base  of forelimb.  ×Heart- pericardium/ atrial wall- stronger on  one side. |
| Wnt10b  n=15 (30) | Mandible primordium, tongue [49], limb in En-/- [26], surface ectoderm restricted [51], AER and mammary ridge [36, 52] | **◇**Several localised regions in facial region-  ectoderm of nasal pits, restricted regions of  maxillary, mandibular and 2^nd^ BA ectoderm  **◇**AER not full extent – more anterior than  posterior  ×Ectoderm at base of limb bud, especially  anterior of forelimb, patches at anterior and  posterior on ventral aspect. Ventral ectoderm  of hindlimb |
| Wnt11  n=5 (15) | Limb buds, branchial arch, nasal process [52], above and somites at Ts17 [53], ureteric bud [53, 54] | ×Head mesenchyme between hindbrain and  forebrain levels- two columns  ×Two columns of expression in mid part of  hindbrain (in transverse section)- at level of  OV  ×Ventricular surface of midbrain and forebrain  × Infundibular recess  ×Mesenchyme around the eyes  **◇**Localised patches of mesenchyme in BAs  ×Tracts running DV at base of BAs -position of  phrenic nerve  **◇**Lateral mesenchyme extending into -4^th^  BA  ×Localised within OV- cochlear primordium  ×Most anterior dorsal root ganglia  ×AER  ×Lateral mesenchyme patch at base of heart-  level of very anterior of forelimb  ×Lateral line between limbs  ×Ventrolateral line along the edge of the  posterior trunk and tail –posterior to hindlimb  buds  ×Pericardium and body wall around heart  ×Notochord and ventral neural tube in tail  **◇** Dermamyotome |
| Wnt16  n=4 (20) | Limb handplate mesenchyme [22] (our results show very lightly stained limb expression in only one stage matched Ts19 specimen- seen in Ts20 and much more clearly at Ts21) | ×Two elongated domains lateral to the  telencephalic vesicles, close to surface but not  ectodermal  ×Localised domains of forebrain, both  diencephalon and telencephalon  ×OV especially ventral cochlear region  ×Outflow tract and heart  ×Around part of midgut within umbilical hernia.  ×Mesenchyme around hindgut  ×Premuscle masses from about somite 10  (level of anterior liver) and more posterior to  about level somite 23. very lateral extent of  Dermamyotome – close to start of hindlimb  bud in T section- from level of somite 20-  extending more throughout dermamyotome as  moves posteriorly.  ×Tail bud- throughout most caudal somites |

Abbreviations: OV; otic vesicle, LPM; lateral plate mesenchyme, BA; branchial arch DRG; dorsal

root ganglia.

1. Wilkinson, D.G., J.A. Bailes, and A.P. McMahon, *Expression of the proto-oncogene int-1 is restricted to specific neural cells in the developing mouse embryo.* Cell, 1987. **50**(1): p. 79-88.

2. Bally-Cuif, L., et al., *Relationship between Wnt-1 and En-2 expression domains during early development of normal and ectopic met-mesencephalon.* Development, 1992. **115**(4): p. 999-1009.

3. Dymecki, S.M. and H. Tomasiewicz, *Using Flp-recombinase to characterize expansion of Wnt1-expressing neural progenitors in the mouse.* Dev Biol, 1998. **201**(1): p. 57-65.

4. McMahon, J.A. and A.P. McMahon, *Nucleotide sequence, chromosomal localization and developmental expression of the mouse int-1-related gene.* Development, 1989. **107**(3): p. 643-50.

5. Monkley, S.J., et al., *Targeted disruption of the Wnt2 gene results in placentation defects.* Development, 1996. **122**(11): p. 3343-53.

6. Bellusci, S., et al., *Evidence from normal expression and targeted misexpression that bone morphogenetic protein (Bmp-4) plays a role in mouse embryonic lung morphogenesis.* Development, 1996. **122**(6): p. 1693-702.

7. Grove, E.A., et al., *The hem of the embryonic cerebral cortex is defined by the expression of multiple Wnt genes and is compromised in Gli3-deficient mice.* Development, 1998. **125**(12): p. 2315-25.

8. Lin, Y., et al., *Induction of ureter branching as a response to Wnt-2b signaling during early kidney organogenesis.* Dev Dyn, 2001. **222**(1): p. 26-39.

9. Burns, R.C., et al., *Requirement for fibroblast growth factor 10 or fibroblast growth factor receptor 2-IIIb signaling for cecal development in mouse.* Dev Biol, 2004. **265**(1): p. 61-74.

10. Ozaki, H., et al., *Six1 controls patterning of the mouse otic vesicle.* Development, 2004. **131**(3): p. 551-62.

11. Roelink, H. and R. Nusse, *Expression of two members of the Wnt family during mouse development--restricted temporal and spatial patterns in the developing neural tube.* Genes Dev, 1991. **5**(3): p. 381-8.

12. Barrow, J.R., et al., *Ectodermal Wnt3/beta-catenin signaling is required for the establishment and maintenance of the apical ectodermal ridge.* Genes Dev, 2003. **17**(3): p. 394-409.

13. Salinas, P.C. and R. Nusse, *Regional expression of the Wnt-3 gene in the developing mouse forebrain in relationship to diencephalic neuromeres.* Mech Dev, 1992. **39**(3): p. 151-60.

14. Salinas, P.C., et al., *Maintenance of Wnt-3 expression in Purkinje cells of the mouse cerebellum depends on interactions with granule cells.* Development, 1994. **120**(5): p. 1277-86.

15. Sarkar, L. and P.T. Sharpe, *Expression of Wnt signalling pathway genes during tooth development.* Mech Dev, 1999. **85**(1-2): p. 197-200.

16. Vacalla, C.M. and T. Theil, *Cst, a novel mouse gene related to Drosophila Castor, exhibits dynamic expression patterns during neurogenesis and heart development.* Mech Dev, 2002. **118**(1-2): p. 265-8.

17. Yoshida, M., et al., *Emx1 and Emx2 functions in development of dorsal telencephalon.* Development, 1997. **124**(1): p. 101-11.

18. Keegan, C.E., et al., *Urogenital and caudal dysgenesis in adrenocortical dysplasia (acd) mice is caused by a splicing mutation in a novel telomeric regulator.* Hum Mol Genet, 2005. **14**(1): p. 113-23.

19. Takada, S., et al., *Wnt-3a regulates somite and tailbud formation in the mouse embryo.* Genes Dev, 1994. **8**(2): p. 174-89.

20. Stark, K., et al., *Epithelial transformation of metanephric mesenchyme in the developing kidney regulated by Wnt-4.* Nature, 1994. **372**(6507): p. 679-83.

21. Miyamoto, N., et al., *Defects of urogenital development in mice lacking Emx2.* Development, 1997. **124**(9): p. 1653-64.

22. Guo, X., et al., *Wnt/beta-catenin signaling is sufficient and necessary for synovial joint formation.* Genes Dev, 2004. **18**(19): p. 2404-17.

23. Heikkila, M., et al., *Wnt-4 deficiency alters mouse adrenal cortex function, reducing aldosterone production.* Endocrinology, 2002. **143**(11): p. 4358-65.

24. Lickert, H., et al., *Expression patterns of Wnt genes in mouse gut development.* Mech Dev, 2001. **105**(1-2): p. 181-4.

25. Yamaguchi, T.P., et al., *A Wnt5a pathway underlies outgrowth of multiple structures in the vertebrate embryo.* Development, 1999. **126**(6): p. 1211-23.

26. Cygan, J.A., R.L. Johnson, and A.P. McMahon, *Novel regulatory interactions revealed by studies of murine limb pattern in Wnt-7a and En-1 mutants.* Development, 1997. **124**(24): p. 5021-32.

27. Parr, B.A., et al., *Mouse Wnt genes exhibit discrete domains of expression in the early embryonic CNS and limb buds.* Development, 1993. **119**(1): p. 247-61.

28. Gavin, B.J., J.A. McMahon, and A.P. McMahon, *Expression of multiple novel Wnt-1/int-1-related genes during fetal and adult mouse development.* Genes Dev, 1990. **4**(12B): p. 2319-32.

29. De Langhe, S.P., et al., *Dickkopf-1 (DKK1) reveals that fibronectin is a major target of Wnt signaling in branching morphogenesis of the mouse embryonic lung.* Dev Biol, 2005. **277**(2): p. 316-31.

30. Chu, E.Y., et al., *Canonical WNT signaling promotes mammary placode development and is essential for initiation of mammary gland morphogenesis.* Development, 2004. **131**(19): p. 4819-29.

31. Heller, R.S., et al., *Expression patterns of Wnts, Frizzleds, sFRPs, and misexpression in transgenic mice suggesting a role for Wnts in pancreas and foregut pattern formation.* Dev Dyn, 2002. **225**(3): p. 260-70.

32. Ang, S.J., et al., *Spatial and temporal expression of Wnt and Dickkopf genes during murine lens development.* Gene Expr Patterns, 2004. **4**(3): p. 289-95.

33. Yang, Y., et al., *Wnt5a and Wnt5b exhibit distinct activities in coordinating chondrocyte proliferation and differentiation.* Development, 2003. **130**(5): p. 1003-15.

34. Gofflot, F., M. Hall, and G.M. Morriss-Kay, *Genetic patterning of the developing mouse tail at the time of posterior neuropore closure.* Dev Dyn, 1997. **210**(4): p. 431-45.

35. Vainio, S., et al., *Female development in mammals is regulated by Wnt-4 signalling.* Nature, 1999. **397**(6718): p. 405-9.

36. Veltmaat, J.M., et al., *Identification of the mammary line in mouse by Wnt10b expression.* Dev Dyn, 2004. **229**(2): p. 349-56.

37. Itaranta, P., et al., *Wnt-6 is expressed in the ureter bud and induces kidney tubule development in vitro.* Genesis, 2002. **32**(4): p. 259-68.

38. Ikeya, M. and S. Takada, *Wnt signaling from the dorsal neural tube is required for the formation of the medial dermomyotome.* Development, 1998. **125**(24): p. 4969-76.

39. Hanks, M.C., et al., *Drosophila engrailed can substitute for mouse Engrailed1 function in mid-hindbrain, but not limb development.* Development, 1998. **125**(22): p. 4521-30.

40. Kim, A.S., et al., *Pax-6 regulates expression of SFRP-2 and Wnt-7b in the developing CNS.* J Neurosci, 2001. **21**(5): p. RC132.

41. Theil, T., et al., *Wnt and Bmp signalling cooperatively regulate graded Emx2 expression in the dorsal telencephalon.* Development, 2002. **129**(13): p. 3045-54.

42. Shu, W., et al., *Wnt7b regulates mesenchymal proliferation and vascular development in the lung.* Development, 2002. **129**(20): p. 4831-42.

43. Niederreither, K., et al., *Retinoic acid synthesis and hindbrain patterning in the mouse embryo.* Development, 2000. **127**(1): p. 75-85.

44. Bouillet, P., et al., *A new mouse member of the Wnt gene family, mWnt-8, is expressed during early embryogenesis and is ectopically induced by retinoic acid.* Mech Dev, 1996. **58**(1-2): p. 141-52.

45. Richardson, M., et al., *Mouse Wnt8B is expressed in the developing forebrain and maps to chromosome 19.* Mamm Genome, 1999. **10**(9): p. 923-5.

46. Lako, M., et al., *A novel mammalian wnt gene, WNT8B, shows brain-restricted expression in early development, with sharply delimited expression boundaries in the developing forebrain.* Hum Mol Genet, 1998. **7**(5): p. 813-22.

47. Lan, Y., et al., *Expression of Wnt9b and activation of canonical Wnt signaling during midfacial morphogenesis in mice.* Dev Dyn, 2006. **235**(5): p. 1448-54.

48. Blackshaw, S., et al., *Genomic analysis of mouse retinal development.* PLoS Biol, 2004. **2**(9): p. E247.

49. Dassule, H.R. and A.P. McMahon, *Analysis of epithelial-mesenchymal interactions in the initial morphogenesis of the mammalian tooth.* Dev Biol, 1998. **202**(2): p. 215-27.

50. Narita, T., et al., *Wnt10a is involved in AER formation during chick limb development.* Dev Dyn, 2005. **233**(2): p. 282-7.

51. Davenport, T.G., L.A. Jerome-Majewska, and V.E. Papaioannou, *Mammary gland, limb and yolk sac defects in mice lacking Tbx3, the gene mutated in human ulnar mammary syndrome.* Development, 2003. **130**(10): p. 2263-73.

52. Christiansen, J.H., et al., *Murine Wnt-11 and Wnt-12 have temporally and spatially restricted expression patterns during embryonic development.* Mech Dev, 1995. **51**(2-3): p. 341-50.

53. Kispert, A., et al., *Proteoglycans are required for maintenance of Wnt-11 expression in the ureter tips.* Development, 1996. **122**(11): p. 3627-37.

54. Majumdar, A., et al., *Wnt11 and Ret/Gdnf pathways cooperate in regulating ureteric branching during metanephric kidney development.* Development, 2003. **130**(14): p. 3175-85.
