# Supplementary material for: 3D representation of Wnt and Frizzled gene expression patterns in the mouse embryo at embryonic day 11.5 (Ts19)
Source: Gene Expr Patterns. 2008 May;8(5):331–48. doi: 10.1016/j.gep.2008.01.007 (PMC2452985; doi:10.1016/j.gep.2008.01.007)
Supplement: Supplementary Table 2 — Fzd gene expression sites at Theiler stage (Ts) 19. List of previously published sites and extended and novel descriptions in the present study. n = the number of specimens fully reconstructed in 3D (number of embryos hybridised from which the reconstructed specimens were selected as representative). [file mmc2.doc]

**Supplementary Data Table 2: Fzd gene expression sites at Theiler stage (Ts) 19**. List of previously published sites and extended and novel descriptions in the present study. n= the number of specimens fully reconstructed in 3D (number of embryos hybridised from which the reconstructed specimens were selected as representative).

| **Gene** | **Previously published sites**  **Æ Not confirmed here** | × Novel Sites¯ Extended details |
| --- | --- | --- |
| Fzd1  n=3 (6) | No previous report at Ts19  Thymus primordium (Ts20, [1]).  Multiple sites up to E10.5 only [2], presomitic mesoderm, intermediate mesoderm; rostro-medial edge of newly formed somites and ventral border of dermamyotome- extends into adjacent limb bud. Transiently in medio-ventral neural tube (until E9)- optic lens, otic placode, olfactory placode, telencephalic vesicles, mesencephalon, foregut, thyroid primordium and Branchial arches (BAs). | ×Midbrain- dorsal, outer (mantle) layer  ×Telencephalon- largely restricted laterally  ×Hindbrain, lateral stripe just posterior to  midbrain-hindbrain boundary. In the more posterior hindbrain expression is in the ventricular zone with a localised intense patch just dorsal to the floor plate at level  anterior to OV  ×Complex patches in the facial region and  branchial arches including around nasal pits.  Anterior maxillary, posterior mandibular and  2^nd^ BA.  ×Limbs: AER, patches in the limb  mesenchyme. Particularly high at anterior  side. Also patches in mesenchyme at dorsal  aspect of base of limbs, particularly strong  and extensive toward anterior aspect.  ×Eye – anterior walls of lens vesicle, corneal  ectoderm, fine line in ventral part of optic stalk  ×Body wall around heart  ×Around hypato-cardiac vein  ×Midline dorsal ectoderm  ×Myotome and extending into body wall and  limb  ×Lateral line between limbs. |
| Fzd2  n=4 (12) | No previous report at Ts19  Pigmented retinal epithelium, neural retinal epithelium (Ts20, [3]) | No specific expression detected |
| Fzd3  n=3 (6) | No previous report at Ts19  Retina (Ts20, [3])  Yolk sac (Ts18, by PCR, [4]), liver (Ts22, by PCR, [4])  Multiple sites up to E10.5 only [2], dorsal neural tube, faintly in somites,1^st^ BA, olfactory placode, distal region of limb buds. Faintly throughout mesenchyme of entire body wall | ×Throughout midbrain, dorsal diencephalon, -  throughout ventricular zone of hindbrain and  more extensive dorsally; dorsal telencephalic  vesicles-quite extensive; strong in cortical  hem.  ×Neural tube- throughout dorsal part of neural  tube, extending ventrally in ventricular zone,  slightly elevated in ventral mantle layer.  ×Epithelium around nasal pit- particularly in  region of vomeronasal organ.  ×Faintly through limb mesenchyme but more  intense in proximal anterior and ventral base-  patch within proximal, dorsal mesenchyme.  ×Dorsal root ganglia  ×Trigeminal ganglion  ×OV, endolymphatic diverticulum and cochlear  primordium)  ×Eye, optic stalk, outer anterior optic cup,  stronger throughout lens vesicle.  × Ganglion of vagus nerve  × Mesenchyme of body wall- faintly through segmental muscle masses- |
| Fzd4  n=7 (45) | No previous report at Ts19  Pigmented retinal epithelium (Ts20, [3])  Lens pit (Ts17, by PCR, [5]).    Up to E10.5 only [2], ventral telencephalon,- extending later to whole vesicles, weakly in BAs | ×Streaks in mandibular component of 1^st^ BA-  precursor of arch cartilage (Meckel’s),  ×Facial patches around nasal pits –particularly  medial (precartilage of nasal septum)  ×Extensive patches in mesenchyme of 2^nd^ BA-  dorsal and ventral of 2^nd^ arch artery.  Patches extending down into 3^rd^ BA, around  cardinal vein- but lighter  ×Limb; three discrete patches in limb  mesenchyme- distal, dorsal and ventral in  proximal- toward anterior- around skeletal  element- 4^th^ , lighter patch even more  proximal –in dorsal.  ×OV, extensive but more ventral  ×Eye, outer layer of optic cup.  ×Around mid gut in umbilical hernia |
| Fzd5  n=11 (30) | No previous report at Ts19  Neural retinal epithelium (Ts20, [3]). Telencephalon (Ts20), eye + forebrain (Ts17) [6]  Yolk sac (Ts18, by PCR) [4], liver (Ts22, by PCR) [4]  Up to E10.5 only [2], telencephalon, later restricts to optic vesicles- sensory layer of retina, weakly in forming lung bud.  At E9- expressed in ventral telencephalon [7] | × Ventricular layer of midbrain and throughout  the ventral aspect of forebrain and also dorsal  part of posterior forebrain, throughout except  part of the lateral telencephalic vesicles.  ×Throughout OV  **◇**In late Ts19/Ts 20 neural retina in region of  the base of the optic stalk. |
| Fzd6  n=2 (6) | No previous report at Ts19  Retina (Ts20, [3])  Up to E10.5 only [2], E8.5 notochord, restricted later to level of presomitic mesoderm, rostro-lateral edge of newly formed somites, from E10.5, line surrounding the forming limb buds, in interlimb region- ectoderm overlying the dermamyotome, foregut and caudally in cloaca, in max and mandibular components of 1^st^ BA, 2^nd^ BA, metanephric duct, metencephalon and ventral midbrain, apical layer of future pigmented retinal tissue [2] | ×Midbrain-hindbrain boundary- complete ring  but not throughout- toward ventral, more  ventricular. Stronger and more extensive on  midbrain side.  ×Ventricular zone of midbrain floor  ×Forebrain: strong in choroid invagination-  especially medial tip of choroid plexus.  Medial aspect of telencephalic vesicles  ×Opening of nasal pits  ×Ectoderm at base of limbs, particularly  ventral- extending into ventral limb ectoderm  ×OV, particularly lateral aspect and region of  endolymphatic diverticulum.  ×Eye- apical/ anterior tip of outer layer of optic  cup  ×Lining of midgut in umbilical hernia  ×Metanephric duct  ×Ventral Trunk ectoderm adjacent to heart  ×Lateral line ectoderm and mammary gland  primordia  ×Most ventrolateral extent of somites in mid  trunk region |
| Fzd7  n=2 (12) | No previous report at Ts19  Lens vesicle and cornea (Ts20, [3])  Yolk sac (Ts18, by PCR, [4]), liver (Ts22, by PCR) [4]  Lens pit, (Ts17) lens vesicle (Ts20 by PCR) [5]  Multiple sites up to E10.5 only [2]; presomitic mesoderm and ventro-lateral borders of newly formed somites, later localised to medial domain of dermamyotome, ventromedial neural tube, otic placode, base of midbrain and strong in hindbrain, mesenchyme of limb and olfactory placode.  E9 neural tube and somites express [7] | ×Ventricular layer in ventral mesencephalon,  strongest at most dorsal extent of the domain  ×Dorso-lateral telencephalic vesicles  ×Two patches about mid way along  diencephalon on d/v axis and toward but not  at ventricular surface.  ×Throughout ventral ventricular of neural tube  ×Most distal mesenchyme of frontonasal  process- clear boundary especially around the  nasal pits. Especially strong anterior medial  mesenchyme around nasal pits. Also distal  mesenchyme in 1^st^ and 2^nd^ BA – but lower.  ×Limbs- through mesenchyme but lowest at  core proximal- elevated patches, strongest in  proximal-ventral, also elevated central distal  and proximal dorsal.  ×Localised in OV- in posterior it is medial and  about half way along AP extent is more lateral  ×Eye, lens vesicle, especially distal/anterior  ×Epithelia of lung buds  ×Viscera, around BA arteries (6^th^) and vitelline  vein  ×Ventral point of urogenital sinus  ×Pancreas primordium  ×Somites – extensive lateral stripes, strongest most dorsally. In cross section the domain is circular surrounding a core of non expression- lateral aspect of muscle mass |
| Fzd8  n=2 (12) | No previous report at Ts19  Retina (Ts20, [3])  Telencephalon (Ts17, Ts20) [6]  Multiple sites up to E10.5 only [2]; somites from E8.5, localised to myotome in more mature, dorso-medial neural tube, telencephalon, diencephalon and transiently in lens placode (E9.5), gut and cloaca, BAs, olfactory placode and in sinus venosus | ×Forebrain- lateral telencephalon- very broad  and strong. Not through cortical hem- in a  restricted domain on ventricular layer of  choroid invagination. In diencephalon  restricted to line of expression adjacent to  telencephalic vesicles along mantle layer.  ×Diencephalon-Midbrain, lateral line  between diencephalon and midbrain-in T  section, stripe of expression ventral to D/V  midline. Dorsal at midbrain/diencephalon  border  ×Hindbrain and neural tube- from midway down  hindbrain and all along NT- ventricular wedge  at mid dorso-ventral point- becomes more  restricted to ventricular at ventral side.  ×Nasal pits- localised epithelium- medial and  deep midline  ×Limbs-complex mesenchyme –patches  ×OV- medial along D/V axis and anterior  ×Eye, light expression on inner side of lens  vesicle, strongest on anterior of optic stalk-  almost all the way around  ×Trigeminal ganglion, nerves running into the  branchial clefts between 1^st^ and 2^nd^ and 2^nd^  and 3^rd^ BAs  ×Somites: very strong expression in long D/V  stripes. In T section the domain runs from  peripheral dorsally to deep ventrally. –very  different to Fzd7  ×Viscera- mesenchyme of umbilical hernia and  mesenchyme around midgut |
| Fzd9  n=13 (30) | Neural retinal epithelium (Ts20, [3])  Telencephalon (Ts17, Ts20) [8]  Restricted to ventral domain of ventricular zone of nervous system [9] with down regulation in the rostral neural tube at E10.5,  **Æ** E11.5 restricted to ventral domain of neural tube ventricular zone. Migrating myotome at E10.5 [9].  Neural tube, trunk skeletal muscle (myotomes), limb skeletal anlagen, craniofacial regions and nephric duct [10].  Up to E10.5 only [2], myotome from E10.5- not newly formed somites, narrower than Fzd8. E8.5 neural tube at somatic level and diencephalon, E10.5 in telencephalon and neural tube- strongly in cervical and caudal regions, faint in BAs [2] | **◇** Telencephalon – cortical hem, base of  choroid plexis  **◇**Neural tube- two ventral domains either side of floor plate in posterior hindbrain. Elsewhere, ventricular at midpoint of neural tube.  **◇**Limb mesenchyme- low level through territory  of future skeletal elements of digits in distal,  concentrated in dorsal mesenchyme in  proximal.  ×OV- elevated in lateral side and cochlear duct |
| Fzd10  n=5 (6) | Telencephalon (Ts20, [8])  Neural tube, somites and Mullerian duct (Ts20, [11]), limb bud also shown at E11.5.  E9 throughout d neural tube,up to diencephalon [7]  **Æ** In polarising zone of chick limb bud [12] | **◇**Throughout dorsal CNS from diencephalon  through neural tube. Very sharp anterior  dorsoventral boundary in diencephalon that  coincides with a neuromere.  ×Lateral surfaces of 1^st^ and 2^nd^ BAs , in lateral  and posterior nasal process but not in anterior  nasal process  **◇**Throughout distal periphery of limb- sections  show both ectoderm and mesenchyme.  ×Lateral line between the limbs –superficial  body wall- along dorsoventral axis at level of  dorsal limb base.  ×Eye- lens vesicle but highest at distal  surface- cornea |

Abbreviations: OV; otic vesicle, CNS:central nervous system, n=number of samples analyzed by OPT, brackets deonte number of embryos in situ hybridized.

1. Bleul, C.C. and T. Boehm, *Laser capture microdissection-based expression profiling identifies PD1-ligand as a target of the nude locus gene product.* Eur J Immunol, 2001. **31**(8): p. 2497-503.

2. Borello, U., et al., *Differential expression of the Wnt putative receptors Frizzled during mouse somitogenesis.* Mech Dev, 1999. **89**(1-2): p. 173-7.

3. Blackshaw, S., et al., *Genomic analysis of mouse retinal development.* PLoS Biol, 2004. **2**(9): p. E247.

4. Austin, T.W., et al., *A role for the Wnt gene family in hematopoiesis: expansion of multilineage progenitor cells.* Blood, 1997. **89**(10): p. 3624-35.

5. Xiao, W., et al., *Gene expression profiling in embryonic mouse lenses.* Mol Vis, 2006. **12**: p. 1692-8.

6. Kim, A.S., D.H. Lowenstein, and S.J. Pleasure, *Wnt receptors and Wnt inhibitors are expressed in gradients in the developing telencephalon.* Mech Dev, 2001. **103**(1-2): p. 167-72.

7. Kemp, C.R., et al., *Expression of Frizzled5, Frizzled7, and Frizzled10 during early mouse development and interactions with canonical Wnt signaling.* Dev Dyn, 2007. **236**(7): p. 2011-9.

8. Kim, A.S., et al., *Pax-6 regulates expression of SFRP-2 and Wnt-7b in the developing CNS.* J Neurosci, 2001. **21**(5): p. RC132.

9. Van Raay, T.J., et al., *frizzled 9 is expressed in neural precursor cells in the developing neural tube.* Dev Genes Evol, 2001. **211**(8-9): p. 453-7.

10. Wang, Y.K., et al., *Characterization and expression pattern of the frizzled gene Fzd9, the mouse homolog of FZD9 which is deleted in Williams-Beuren syndrome.* Genomics, 1999. **57**(2): p. 235-48.

11. Nunnally, A.P. and B.A. Parr, *Analysis of Fz10 expression in mouse embryos.* Dev Genes Evol, 2004. **214**(3): p. 144-8.

12. Kawakami, Y., et al., *Involvement of frizzled-10 in Wnt-7a signaling during chick limb development.* Dev Growth Differ, 2000. **42**(6): p. 561-9.
