# Supplementary material for: 3D representation of Wnt and Frizzled gene expression patterns in the mouse embryo at embryonic day 11.5 (Ts19)
Source: Gene Expr Patterns. 2008 May;8(5):331–48. doi: 10.1016/j.gep.2008.01.007 (PMC2452985; doi:10.1016/j.gep.2008.01.007)
Supplement: Supplementary Table 3 — Supplementary data: details of gene expression probes. [file mmc3.doc]

Table 3 supplementary data: details of gene expression probes

| Gene | Extent of Probe on Genbank Sequence | Source |
| --- | --- | --- |
| Wnt1 | Nucleotide 138 to 2345 on BC005449.1 | A. McMahon |
| Wnt2 | Nucleotide 35 to 1510 on NM_023653.4 | A. McMahon |
| Wnt2b | Nucleotide 1010 to 1215 on NM_009520.3 | L. Zakin |
| Wnt3 | Nucleotide 1609 to 3000 on NM_009521.1 | P. Salinas |
| Wnt3a | Nucleotide 2310 to 2676 on NM_009522.1 | RT-PCR generated |
| Wnt4 | Nucleotide 639 to 1101 on NM_009523.1 | A. McMahon |
| Wnt5a | Nucleotide 576 to 2714 on NM_009524.2 | A. McMahon |
| Wnt5b | Nucleotide 111 to 1429 on NM_009525.2 | A. McMahon |
| Wnt6 | Nucleotide 27 to 2066 on NM_009526.3 | A. McMahon |
| Wnt7a | Nucleotide 30 to 3173 on NM_009527.3 | A. McMahon |
| Wnt7b | Nucleotide 93 to 1581 on NM_009528.2 | A. McMahon |
| Wnt8a | Nucleotide 87 to 1746 on NM_009290.1 | P. Chambon |
| Wnt8b | Nucleotide 939 to 1631 on NM_011720.2 | J. Mason |
| Wnt9a | Nucleotide 1699 to 2246 on NM_139298.2 | RT-PCR generated |
| Wnt9b | Nucleotide 2833 to 3456 on NM_011719.3 | RT-PCR generated |
| Wnt10a | Nucleotide 289 to 2469 on NM_009518.1 | IMAGE clone |
| Wnt10b | From nucleotide 294 on NM_011718.1 plus 3’ and 5’ sequences on NW_001030577.1 (3.2 kb) | A. McMahon |
| Wnt11 | Nucleotide 169 to 1821 on NM_009519.1 plus 376 bp 5’ sequence (NW_001030877.1) | A. McMahon |
| Wnt16 | Nucleotide 537 to 1531 on NM_053116.3 | RT-PCR generated |
| Fzd1 | Nucleotide 2774 to 3663 on NM_021457.2 | RT-PCR generated |
| Fzd2 | Nucleotide 3289 to 3628 on NM_020510.2 plus 361 bp 3’ sequence (NW_001030434.1) | IMAGE clone |
| Fzd3 | Nucleotide 1 to 1321 on NM_021458.1 | U. Borello/G. Cossu |
| Fzd4 | Nucleotide 1394 to 1834 on NM_008055.3 | U. Borello/G. Cossu |
| Fzd5 | Nucleotide 1513 to 1742 on NM_022721.3 | U. Borello/G. Cossu |
| Fzd6 | Nucleotide 1794 to 2642 on NM_008056.2 | U. Borello/G. Cossu |
| Fzd7 | Nucleotide 468 to 1290 on NM_008057.3 | U. Borello/G. Cossu |
| Fzd8 | Nucleotide 968-1965 on NM_008058.1 | U. Borello/G. Cossu |
| Fzd9 | Nucleotide 823 to 1323 on XM_284144.5 | U. Borello/G. Cossu |
| Fzd10 | Nucleotide 1829 to 2988 on NM_175284.3 | IMAGE clone |
